# Constitutional mismatch repair deficiency syndrome with atypical features caused by a homozygous *MLH1* missense variant (c.1918C>A, p.(Pro640Thr)): a case report

**DOI:** 10.3389/fonc.2023.1195814

**Published:** 2023-08-17

**Authors:** Firas Akrout, Ahlem Achour, Carli M. J. Tops, Richard Gallon, Rym Meddeb, Sameh Achoura, Mariem Ben Rekaya, Emna Hamdeni, Soumaya Rammeh, Ridha Chkili, Nada Mansouri, Neila Belguith, Ridha Mrad

**Affiliations:** ^1^ Department of Neurosurgery, Military Hospital of Tunis, Tunis, Tunisia; ^2^ Faculty of Medicine of Tunis, University of Tunis El Manar, Tunis, Tunisia; ^3^ Department of Congenital and Hereditary Diseases, Charles Nicolle Hospital, Tunis, Tunisia; ^4^ Department of Clinical Genetics, Leiden University Medical Center, Leiden, Netherlands; ^5^ Laboratory of Human Genetics, Faculty of Medicine of Tunis, University of Tunis El Manar, Tunis, Tunisia; ^6^ Translational and Clinical Research Institute, Faculty of Medical Sciences, Newcastle University, Newcastle upon Tyne, United Kingdom; ^7^ Research Unit of Onco-theranostic Biomarkers UR17ES15, Faculty of Medicine of Tunis, University of Tunis El Manar, Tunis, Tunisia; ^8^ Department of Pathology, Charles Nicolle Hospital, Tunis, Tunisia; ^9^ Department of Pathology, Military Hospital of Tunis, Tunis, Tunisia; ^10^ Laboratory of Human Molecular Genetics, Faculty of Medicine of Sfax, University of Sfax, Sfax, Tunisia

**Keywords:** CMMRD, hereditary cancer syndromes, next-generation sequencing, MLH1 gene, Lynch syndrome, cMSI, case report

## Abstract

Constitutional mismatch repair deficiency (CMMRD) syndrome is a rare autosomal recessive genetic disorder caused by biallelic germline mutations in one of the mismatch repair genes. Carriers are at exceptionally high risk for developing, typically in early life, hematological and brain malignancies, as well as cancers observed in Lynch syndrome. We report a homozygous *MLH1* missense variant (c.1918C>A p.(Pro640Thr)) in a Tunisian patient with CMMRD syndrome and a family history of early-age colorectal cancer. The proband presented initially with colonic oligopolyposis and adenosquamous carcinoma of the caecum. He later developed several malignancies, including undifferentiated carcinoma of the parotid, grade 4 IDH-mutant astrocytoma, and ampulla of Vater adenocarcinoma. The patient was older than typical for this disease and had a remarkably prolonged survival despite developing four distinct aggressive malignancies. The current report highlights the challenges in assessing the pathogenicity of the identified variant and the remarkable phenotypic diversity in CMMRD.

## 1 Introduction

Constitutional mismatch repair deficiency (CMMRD; MIM 276300) is a recessive childhood cancer syndrome caused by pathogenic variants in both alleles of one of the mismatch repair (MMR) genes (*MLH1*, MIM *120436; *MSH2*, MIM *609309; *MSH6*, MIM *600678 and *PMS2*, MIM *600259) (1). Unlike Lynch syndrome (LS), which is due to monoallelic variants in one of the same genes and leads mainly to colorectal and endometrial cancer development, CMMRD predisposes to a broad tumor spectrum. These malignancies include most frequently hematological, brain, and LS-associated neoplasms. CMMRD is often associated with features suggestive of neurofibromatosis type 1, specifically café-au-lait macules. Consensus reports suggest use of an indication score to identify at-risk patients, genetic testing, and ancillary molecular assays to confirm the diagnosis of CMMRD, and to implement cancer surveillance programs (2, 3). We herein describe a family in which three of the four siblings met the clinical criteria of CMMRD testing. One of them survived long-term, underwent genetic testing, and was found to have a homozygous germline missense variant (c.1918C>A p.(Pro640Thr)) in the *MLH1* gene. We describe the clinical, pathological, and genetic findings of the patient harboring this variant, who exhibits strong evidence supporting the diagnosis of CMMRD and whose phenotype exhibits several original features.

## 2 Case description

**Figure 1:**
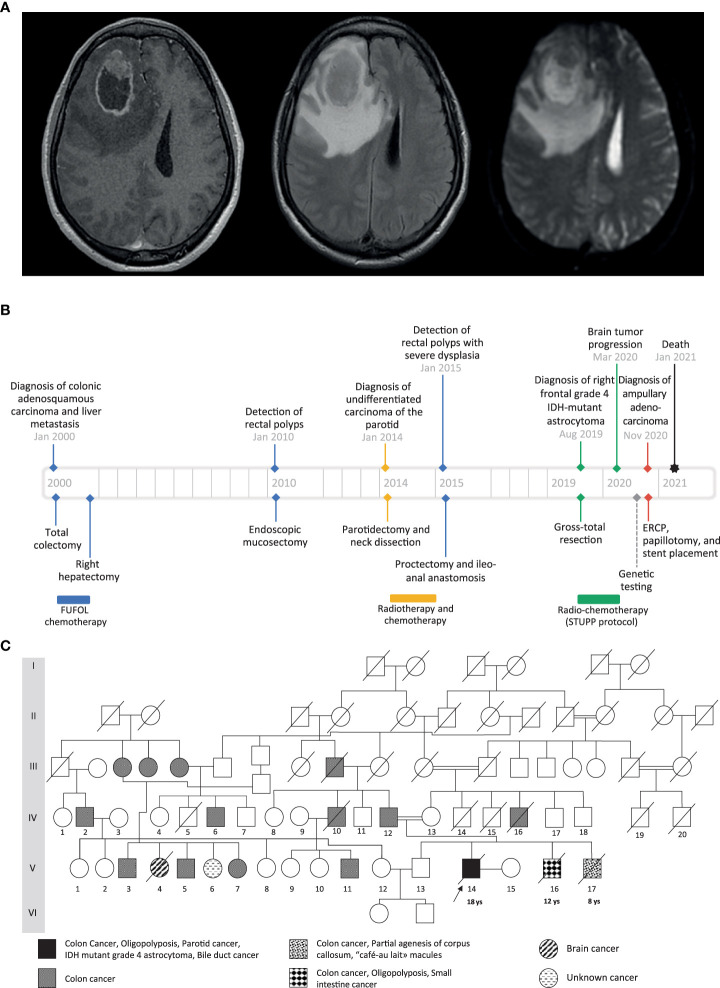
**(A)** Preoperative cranial MRI of the proband (from left to right: axial T1-weighted with gadolinium injection, axial T2 FLAIR-weighted, axial apparent diffusion coefficient). There is a solid intra-axial mass within the right frontal lobe with marked ring-like enhancement and an internal necrotic area. The enhancing component of the lesion shows diffusion restriction. **(B)** Timeline showing the clinical history of the patient. **(C)** Family pedigree. The black arrow indicates the proband case. The age of the first tumor is given below the proband and the two affected brothers.

The proband, a Tunisian male and the child of first cousins, presented at age 18 with a right-sided malignant colonic obstruction for which he underwent a total colectomy. Histopathology classified the tumor as an invasive adenosquamous carcinoma (pT4a, pN0, R0). Four tubulo-villous and tubular adenomatous polyps were also found in the removed specimen. Two of them showed high-grade dysplasia. Chemotherapy was administered according to the FUFOL regimen. Synchronous liver metastasis was identified during the postoperative investigation, and right hepatectomy was performed. At age 32, an undifferentiated carcinoma of the parotid was diagnosed, which was radically resected and locally treated with radiotherapy. One year later, about 20 synchronous rectal adenomas were detected on screening colonoscopy. One of them was severely dysplastic and a proctectomy was performed. At age 37, the patient exhibited headache, vomiting, and left-sided weakness. Imaging revealed a right frontal mass involving the corpus callosum with a marked ring-like gadolinium enhancement, central necrosis, and peritumoral edema (**Figure 1A**). Craniotomy and gross total resection of the tumor were done, and it was diagnosed as grade 4, isocitrate dehydrogenase (IDH)-mutant astrocytoma. He received radiochemotherapy according to Stupp’s protocol. At age 38, he developed obstructive jaundice due to a mass in the head of the pancreas. He underwent endoscopic retrograde cholangiopancreatography followed by papillotomy and placement of a plastic stent in the common bile duct. Histopathological examination revealed an adenocarcinoma of the ampulla of Vater. Three months later, the patient passed away from aggressive local and distant recurrence of his high-grade astrocytoma. A timeline depicting patient care is shown in **Figure 1B**.

Because of the history of metachronous malignancies and consanguinity, a cancer predisposition syndrome was suspected. The proband and his family were referred to genetic consultation, and a six-generation pedigree was established (**Figure 1C**). Physical examination revealed neither café-au-lait spots nor other features of neurofibromatosis type 1. Family history was significant for early-onset colorectal cancer (CRC) in two brothers: One (V.16) had undergone a screening colonoscopy at age 12, and was found to have several dysplastic polyps involving the colon and the small intestine. One of them showed invasive adenocarcinoma requiring a total colectomy. The youngest brother (V.17) was diagnosed with adenocarcinoma of the sigmoid at age 8. He also had a history of partial agenesis of the corpus callosum and café-au-lait macules. Both died from their diseases at ages 18 and 9, respectively. The proband’s father (IV.12) was diagnosed, at age 58, with several highly dysplastic polyps on screening colonoscopy, which led to performing a preventive total colectomy. The eldest brother (V.13) and the mother (IV.13) didn’t present any tumors at ages 41 and 63, respectively. Family history and clinical phenotype were consistent with CMMRD and the proband received a score of 11, meeting the indication criteria for CMMRD testing (2).

**Figure 2:**
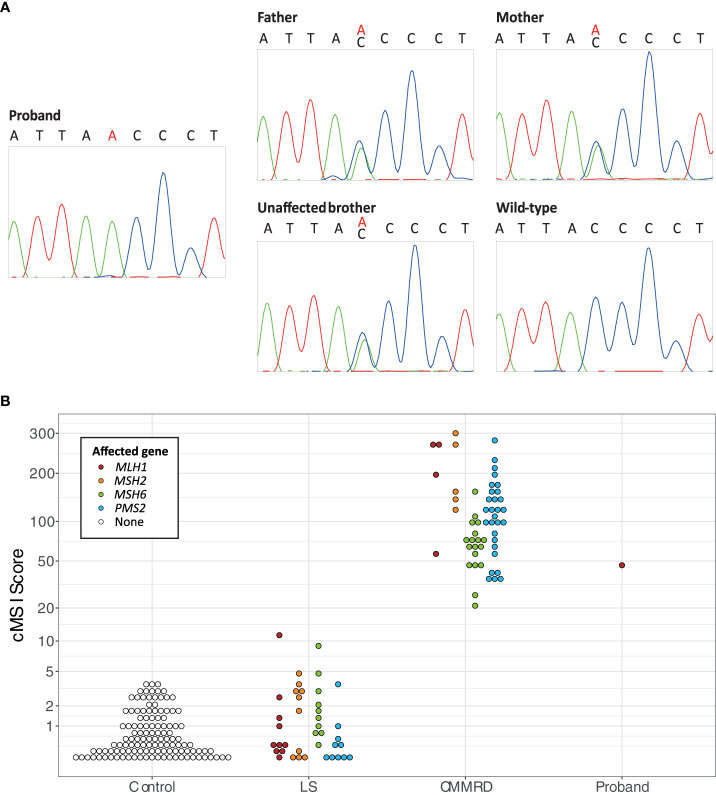
**(A)** Sanger sequencing results of the segregation study of the *MLH1* c.1918C>A variant, including a chromatogram of the proband showing the homozygous *MLH1* c.1918C>A variant, chromatograms of the father, the mother, and the unaffected brother showing the heterozygous *MLH1* c.1918C>A variant, and a chromatogram of the wild type sequence. **(B)** The constitutional MSI (cMSI) scores of 123 control, 40 Lynch syndrome (LS; MLH1 n = 10, MSH2 n = 10, MSH6 n = 10, PMS2 n = 10), 56 constitutional mismatch repair deficiency syndrome (CMMRD; MLH1 n = 4, MSH2 n = 5, MSH6 n = 18, PMS2 n = 29), and 43 control patients using data from Gallon et al. (4), compared to the cMSI score of the proband. The y-axis is scaled based on a logit transformation.

Genomic DNA was extracted from the peripheral blood leukocytes (PBLs) of the proband, unaffected brother, and parents. A CRC predisposition gene panel, including the MMR genes, was analyzed. Next-generation sequencing was performed on an Illumina platform with the Agilent enrichment kit SureSelectXT Clearseq inherited disease Panel. Data analysis was processed for all coding exons, including 20 nucleotides in the flanking intron sequences, with in-house pipelines. Sanger sequencing was performed using Big Dye terminator cycle sequencing Kit v.3.1 on a Seq Studio Applied Biosystems Sequencing platform. Genetic analysis revealed a homozygous variant (c.1918C>A; p.(Pro640Thr)) in the *MLH1* gene: a substitution of cytosine by adenine in exon 17 leading to a missense mutation of a proline to a threonine. The variant was also identified in a heterozygous state in the father, the mother, and the eldest brother (**Figure 2A**). Unfortunately, the two deceased brothers have not been tested because DNA and tissue samples were unavailable.

The *MLH1* c.1918C>A variant was not found in gnomAD (https://gnomad.broadinstitute.org/) and was predicted to be damaging by in silico software, including Polyphen2 (HumVar score 0.999) (http://genetics.bwh.harvard.edu/pph2/), SIFT (Score 0.00) (https://sift.bii.a-star.edu.sg/), AlignGVGD (Class C35) (http://agcgd.hci.utah.edu), and CADD (Phred score 26.7, Raw score 3.86) (http://cadd.gs.washington.edu/). The MAPP-MMR + Polyphen-2 prior probability for pathogenicity was 0.83 (http://priors.hci.utah.edu/PRIORS/) (5). *MLH1* c.1918C>A has recently been observed as a heterozygous variant in several families with an LS phenotype. It was also shown to disrupt protein expression in a transfected HEK293T cell line model, but had minimal effect on MMR activity in vitro (6). In contrast, Takahashi et al. (7) had previously found >75% expression of MLH1 protein containing the variant in a transfected HCT116 cell line model, but an approximate 50% reduction in repair activity in vitro. These data suggest homozygosity of *MLH1* c.1918C>A may cause CMMRD in the proband, but further investigations were pursued to confirm the diagnosis given the conflicting functional test results.

**Figure 3:**
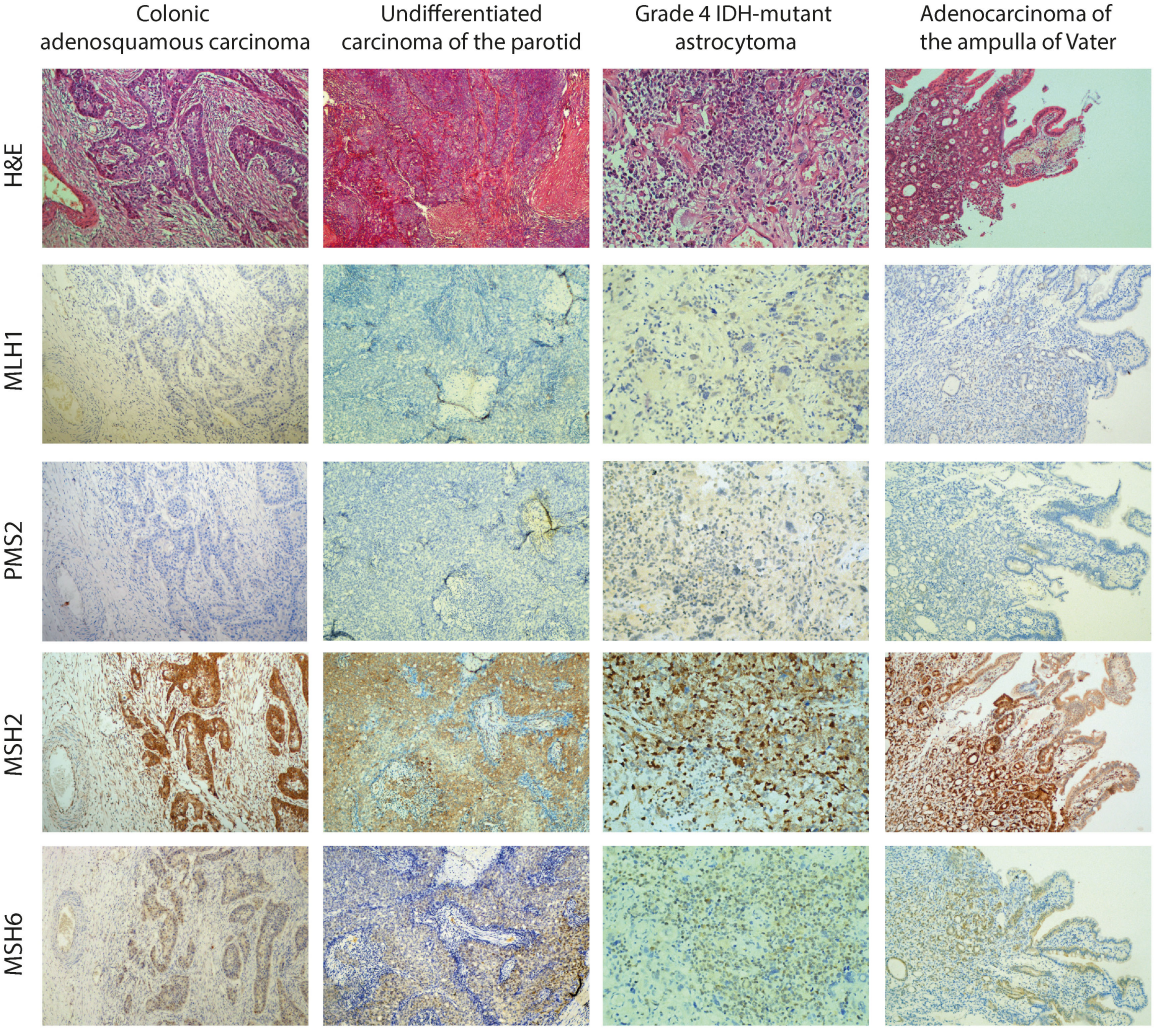
Histology (hematoxylin and eosin (H&E)) and immunohistochemical staining for all four MMR proteins (MLH1, PMS2, MSH2, and MSH6) in all tumor specimens of the proband (colonic and parotid tumor at 40× magnification, brain tumor and pancreatic tumor at 100× magnification). As the representative images show, immunostaining for MLH1 and PMS2 revealed a loss of expression in neoplastic cells and surrounding normal cells.

Immunohistochemistry staining for all four MMR proteins was assessed for all tumor specimens of the proband. We used an external positive control tissue from another individual for each antibody stain. The microscopic examination showed loss of MLH1 and PMS2 expression in neoplastic cells and surrounding normal cells, which is consistent with but not confirmatory of a CMMRD diagnosis caused by variants in *MLH1*. Immunostaining was retained for MSH2 and, to a lesser extent, for MSH6 (**Figure 3**).

Microsatellite instability (MSI) analysis was performed on DNA samples extracted from formalin fixed paraffin embedded tissue of the colorectal, cerebral, parotid, and ampulla of Vater tumors, and non-tumoral colorectal mucosa. Six monomorphic microsatellite markers (NR21, NR24, NR27, BAT25, BAT26 and HSP110) were amplified and the amplicons were separated and visualized using the ABI Seq Studio Genetic Analyzer (Applied Biosystem). Fragment length analysis of microsatellite amplicons showed instability in both tumoral and non-tumoral colorectal tissues, as well as in the cerebral, parotid, and ampulla of Vater tumors. However, in blood, all MSI markers showed a stable profile (**Supplementary Table 1**).

Along with tumor analyses, the impact of *MLH1* c.1918C>A; p.(Pro640Thr) on MMR function was further assessed using the cell-free in vitro MMR activity (CIMRA) assay (8) and was shown to have 17% relative MMR activity. Together, these results provide strong evidence for the pathogenicity of *MLH1* c.1918C>A (**Table 1**) and support a diagnosis of CMMRD in the proband.

**Table 1:** *MLH1* c.1918C>A; p.(Pro640Thr) variant interpretation according to ClinGen InSiGHT Hereditary Colorectal Cancer/Polyposis Variant Curation Expert Panel Specifications to the ACMG/AMP Variant Interpretation Guidelines Version 1.

InSiGHT Criteria	Criteria description	Met/not Met
PS3_Strong	CIMRA test 17% relative MMR^1^ (8) Deficient protein function and expression similar to a reference pathogenic variant based on lab assay (6)	Met^a^
PM2_Supporting	Absent/extremely rare (<1 in 50,000 alleles) in gnomAD using the non-cancer dataset.	Met^b^
PM3_Supporting	Homozygous occurrence in the same gene in a patient with clinical features consistent with CMMRD and/or documented MMR deficiency in normal cells	Met^c^
PP3_Moderate	Missense variant with MAPP+PolyPhen-2 prior probability for pathogenicity >0.81	Met^d^
PP4_Moderate	Two independent tumors with MSI and/or loss of MMR protein expression consistent with the variant location	Met^e^

^1^ Tested and calculated as described by Drost et al. (8).

a The two criteria are met.

b Variant is absent in gnomAD.

c Evidence strength for homozygous occurrence 0.5.

d Prior probability for pathogenicity 0.83 (http://priors.hci.utah.edu/PRIORS/).

e Loss of MLH1 expression in colonic, parotid, ampulla of Vater, and brain tumors.

Variant interpretation according to rules for combining pathogenic criteria ACMG/AMP: 1 Strong + 2 Moderate + 2 Supporting = Pathogenic.

As a final confirmation of the diagnosis, PBL DNA of the proband was analyzed for constitutional MSI (cMSI), MSI in non-neoplastic tissues that is a highly specific molecular feature of CMMRD. cMSI is not detectable by methods designed for tumor MSI analysis and requires specialist assays (4). The cMSI assay amplifies and sequences 32 highly sensitive MSI markers, and uses the frequency of microsatellite insertion and deletion variants to calculate a cMSI score for each sample. The higher the cMSI score the higher the constitutional burden of MSI. The proband had a cMSI score of 44.3, which falls within the published cMSI score range of known CMMRD patients (20.9-300.0) and is much greater than the cMSI scores of LS carriers (0.0-11.3) and controls (0.0-3.6) (4). cMSI analysis, therefore, confirmed the CMMRD diagnosis (**Figure 2B**).

## 3 Discussion and conclusions

Biallelic germline variants in MMR genes are known to cause the recessive CMMRD syndrome. The latter was recognized as a distinct childhood cancer predisposition syndrome in 1999, although it was initially named Turcot syndrome (9, 10). After its first description, more than a hundred cases of biallelic germline variants in one of the MMR genes, including *PMS2*, *MSH2*, *MSH6*, and *MLH1*, have been identified (11). The c.1918C>A; p.(Pro640Thr) variant of the *MLH1* gene identified in our patient has been previously detected in LS (6). However, to our knowledge, a biallelic case of *MLH1* c.1918C>A; p.(Pro640Thr) has never been reported in CMMRD syndrome. Many pieces of evidence were suggestive of the pathogenicity of this variant and its involvement in our patient’s phenotype. Apart from its absence from population databases (PM2_Supporting), it is predicted to be damaging (Prior probability of pathogenicity 0.83) (PP3_Moderate) and is located in a conserved interaction domain between *MLH1* and *PMS2* 506-675 (12). Recently, Mahdouani et al. reported *MLH1* c.1918C>A; p.(Pro640Thr) heterozygotes in three Tunisian families with an LS phenotype from the same geographical area of our proband. They compared protein stability and catalytic activity between the MLH1 p.(Pro640Thr) variant and a validated reference pathogenic variant MLH1 p.(Ala681Thr). The p.(Pro640Thr) variant results in severe defects in protein stability and minimal reduction in catalytic activity (6) (PS3_Strong). However, due to conflicting functional results from Takahashi et al., we conducted further assessment of the functional impact of the variant.

IHC showed loss of MLH1 and PMS2 expression in all tumor and normal cells within tumor tissue sections, consistent with a CMMRD diagnosis. MLH1 and PMS2 are known to act as partners in the repair process, and so functional defects in MLH1 can lead to the instability of both MLH1 and PMS2. Therefore, the IHC results were consistent with the functional analyses of Mahdouani et al., who found the missense variant p.(Pro640Thr) reduced MLH1 protein expression (6). MSI was identified in both non-neoplastic and neoplastic colorectal tissue, and found MLH1 p.(Pro640Thr) caused reduced MMR activity by CIMRA (8). According to the ACMG-adapted InSiGHT criteria (under review, Draft InSiGHT ACMG MMR gene variant classification criteria version 1; https://www.insight-group.org/criteria/), these findings allowed us to classify the variant as pathogenic (**Table 1**).

The variant was also identified in the heterozygous state in the father, who had a history of CRC consistent with LS, and in the unaffected mother and brother, whose lack of cancer history can be explained by the incomplete penetrance of the LS phenotype. The two brothers who died from their disease could not be tested. However, we speculate that they were homozygous for the *MLH1* c.1918C>A variant given their ages at diagnosis and the severity of their phenotypes.

MSI was observed in tumor tissues and in colorectal mucosa of the proband but not in their PBLs using PCR and fragment length analysis of six mononucleotide repeat MSI markers. Several parameters influence MSI assay sensitivity, such as the structure of the repetitive motif (di- or mono-nucleotide repeat), the number of used markers, and the analysis method. Moreover, MSI is the consequence of MMR deficiency during DNA replication. We, therefore, hypothesize that MSI detection depends on the rate of cell division and the lifespan of the cells in each tissue, which could explain the MSS profile detected in our patient’s non-neoplastic blood cells as compared to the MSI profile of the colorectal mucosa. The MSI score was the lowest in the cerebral tumor and highest in the colorectal mucosa and tumor tissues, as well as parotid and ampulla of Vater tumors (**Supplementary Table 1**). Previous studies reported that MSI often fails in brain malignancies and it has been suggested that IHC in all malignant brain tumors younger than 25 years old could be used to screen for CMMRD (13).

Detection of MSI in the constitutional tissues (cMSI) of CMMRD patients requires specialist techniques. For example, Bodo et al. found that PCR and fragment length analysis of mononucleotide repeat MSI markers could not detect MSI in the PBLs but could detect MSI in the immortalized lymphoblastoid cell lines (cultured to develop the MSI signal) of CMMRD patients – a technique named ex vivo MSI analysis (14). More recently, several studies have shown the utility of next-generation sequencing for the detection of cMSI directly from CMMRD PBLs, using either targeted amplicon sequencing or whole genome sequencing approaches (15–17). Here, we used a published amplicon sequencing-based cMSI assay of 32 mononucleotide repeat MSI markers that had been selected from genome sequencing of CMMRD PBL samples specifically for cMSI analysis, which is highly sensitive and specific for CMMRD detection (4). The proband’s cMSI score of 44.3 corroborates a diagnosis of CMMRD and classification of *MLH1* c.1918C>A; p.(Pro640Thr) as pathogenic. Interestingly, a cMSI score of 44.3 is the lowest observed for CMMRD caused by MLH1 deficiency but is consistent with previous observations that missense MMR gene variants are associated with lower cMSI scores (4). Despite observed correlations between MMR genotype and cMSI phenotype, no significant association of cMSI score with age at first cancer was previously found (4). Here, it would have been of interest to compare cMSI scores of the proband and their two affected brothers, who we assume shared a homozygous *MLH1* c.1918C>A genotype, given their very different ages at first cancer, but DNA was not available from the brothers for genetic or cMSI analyses.

CMMRD is poorly recognized by clinicians, and its diagnosis tends to be delayed due to the lack of specific clinical features and the broad tumor spectrum. To address this problem, the European consortium “Care for CMMRD” suggested a scoring system to help physicians to diagnose this disease at the time of the first malignancy (2). According to this scoring system, our patient would have reached a high enough score at the time of his first tumor to initiate CMMRD testing. However, he was suspected of suffering from CMMRD only when he developed his third malignancy. Hence, clinicians should be aware of this cancer predisposition syndrome as a rare cause of early-onset malignancy to achieve timely diagnosis and thus provide an appropriate surveillance program and genetic counseling.

Our case supplements the existing literature by illustrating several atypical features. First, the patient had an atypical course of his disease, with the development of initial cancer at a relatively older age and a remarkably prolonged survival despite developing four distinct aggressive malignancies. In 2014, Wimmer and Kratz reviewed all reported cases, including 146 individuals with CMMRD syndrome, and 82% were younger than 18 years of age at the time of diagnosis of their first malignancy (2). Hematological malignancies are reported to occur in early childhood, around age 5 years, whereas brain cancers develop later with a median age at diagnosis of 9 years, and LS-associated tumors, mostly CRC, develop even later with a median age at diagnosis of 17 years. In our case, the patient presented initially at 18 years of age with colonic cancer. This suggests that CMMRD syndrome is not confined to children and may also manifest in early adulthood. Long-term survival in CMMRD syndrome is also uncommon due to a particularly poor prognosis, with median overall survival of 27 months after the diagnosis of the first malignancy (18). Studies suggest that prognosis varies depending on which MMR gene is affected. Indeed, individuals with biallelic *MLH1* or *MSH2* variants show a more severe phenotype and a lower chance of surviving the first tumor than those with biallelic *MSH6* or *PMS2* variants (1). Surprisingly, the proband in the present study lived 20 years after his first cancer diagnosis, and developed three other malignancies. His relatively long lifespan contrasts with the variant occurring in the *MLH1* gene rather than other MMR genes. Previous cases of attenuated CMMRD have been reported, characterized most frequently by CRC in early adulthood rather than hematological malignancies or brain tumors, caused by a hypomorphic splice site variant in *PMS2* (19). These cases have also been analyzed by the cMSI assay used here and, similar to the proband, had relatively low cMSI scores (4). This, the functional results of Mahdouani et al. (6), and the atypical phenotype of the proband suggest the *MLH1* c.1918C>A; p.(Pro640Thr) could also be considered hypomorphic. Alternatively, the relatively mild phenotype of the proband may be explained by other genetic or environmental factors, and the effective management of each tumor, including radical treatment of his cancers and prophylactic proctectomy to avoid additional rectal malignancies.

Second, the vast majority of high-grade astrocytomas in CMMRD patients are IDH-wildtype (glioblastoma), and IDH-mutant astrocytoma, as diagnosed in the proband, rarely occurs. IDH-wildtype and -mutant tumors are two distinct subsets of high-grade astrocytomas that develop through different genetic pathways and exhibit dissimilar prognoses (20). In a histological review of 26 grade 4 astrocytomas in the setting of CMMRD syndrome, all tumors were identified as IDH-wildtype except for one (21). We report another rare case with grade 4 IDH-mutant astrocytoma. The current standard treatment for grade 4 astrocytoma involves maximal surgical resection, followed by radiotherapy and temozolomide chemotherapy (22). However, MMR-deficient high-grade astrocytomas are known to be resistant to temozolomide but immunotherapy has shown promising results (21, 23). Unfortunately, our index patient was diagnosed with CMMRD syndrome after undergoing temozolomide treatment, which could explain the observed tumor resistance to this methylating drug. Thus, timely diagnosis of CMMRD syndrome is necessary not only for pre-emptive cancer surveillance but also for choosing the most effective therapeutic regimen.

Third, our patient developed an undifferentiated carcinoma of the parotid and an adenocarcinoma of the ampulla of Vater, two unusual malignancies that have been reported only once or twice in CMMRD syndrome. The European Consortium Care for CMMRD divided CMMRD tumors into four groups: hematological malignancies, central nervous system tumors, LS-associated tumors, and other unusual neoplasms. Several tumors belonging to the latter group were described in isolated CMMRD cases, including neuroblastoma, Wilms tumors, rhabdomyosarcoma, ovarian neuroectodermal tumor, infantile myofibromatosis, breast cancer, sarcoma, and pilomatricoma (2). Parotid cancer was not listed but had been separately reported by Baas et al. in a child carrying a biallelic *PMS2* variant, who had a history of B-cell Hodgkin lymphoma, agenesis of the corpus callosum, and developed at age 11 a mucoepidermoid carcinoma of the parotid gland (24). To our knowledge, our case is the second report of a malignant salivary gland tumor in CMMRD. Pancreaticobiliary tumors are also unusual in CMMRD syndrome, although these are included in the spectrum of LS-associated malignancies (25). A carcinoma of the papilla, diagnosed at age 22, has been described in one CMMRD case carrying a biallelic *PMS2* variant (26), and an ampullary adenocarcinoma, diagnosed at age 12, has been described in another carrying a biallelic *MSH6* variant (27). To our knowledge, our proband is the third reported case of pancreaticobiliary cancer in a CMMRD patient. Small intestine cancer is often observed in individuals with CMMRD syndrome and belongs to the LS-associated tumors group (28, 29). This was the case in the proband’s brother, who developed small bowel cancer at age 17 and was likely homozygous for the *MLH1* c.1918C>A variant.

Finally, the proband presented with colonic oligopolyposis reminiscent of attenuated familial adenomatous polyposis or *MUTYH*-associated polyposis. Colonic polyposis is absent in LS, and polyps in this “hereditary non-polyposis syndrome” are only slightly more prevalent than in the general population (30). However, oligopolyposis is a common finding in CMMRD syndrome and can cause misdiagnosis of CMMRD patients as having attenuated familial adenomatous polyposis (26). Therefore, CMMRD syndrome should be considered in patients with childhood-onset adenomatous polyposis in the absence of proven *APC* or *MUTYH* germline variants, as suggested by Herkert et al. and argued by expert opinion from the international CMMRD Consortium (2, 31).

In conclusion, the CMMRD case described here contributes to the continuing evolution of our knowledge of the CMMRD syndrome. Molecular diagnosis allowed accurate genetic counseling for this family, with predictive genetic testing being proposed to facilitate targeted preventive surveillance. Additional assessment of the functional impact of *MLH1* c.1918C>A; p.(Pro640Thr) and the detection of increased cMSI in the proband has confirmed its pathogenicity. The incidence of the *MLH1* c.1918C>A variant in Tunisian families with LS and CMMRD could be consistent with a founder effect. However, more studies and enlarging our cohort are needed to confirm this hypothesis. Our report of a CMMRD patient, diagnosed with parotid and pancreaticobiliary malignancies, provides an unusual phenotypic expression of biallelic MMR gene variant carriers and broadens the tumor spectrum observed in this condition. Even though CMMRD syndrome is exceptionally rare, increasing awareness of this disease is crucial, not only to implement cancer surveillance and prophylaxis but also to deliver accurate and effective cancer therapy.

## Data availability statement

The original contributions presented in the study are included in the article/**Supplementary Material**. Further inquiries can be directed to the corresponding author.

## Ethics statement

The studies involving humans were approved by Ethics Committee of Charles Nicolle Hospital (FWA00032748). The studies were conducted in accordance with the local legislation and institutional requirements. The participants provided their written informed consent to participate in this study. Written informed consent was obtained from the individual(s) for the publication of any potentially identifiable images or data included in this article.

## Author contributions

FA and AA conceived and drafted the manuscript. CT, NB, and AA contributed to the genomic data analysis and interpretation. RG performed cMSI analysis and contributed sections to the manuscript. NB contributed to genetic counseling. SR, MB, and EH performed MSI testing and contributed sections to the manuscript. CT, NB, RyM, and RiM critically revised the manuscript for important intellectual content. FA, SA and RC participated in the patient’s clinical care and contributed intellectually. SR and NM interpreted immunohistochemistry data and provided a pathological assessment of the case. FA and AA authors contributed equally to this work. All authors read and approved the final manuscript.
