# Supplementary material for: Constitutional mismatch repair deficiency syndrome with atypical features caused by a homozygous MLH1 missense variant (c.1918C>A, p.(Pro640Thr)): a case report
Source: Front Oncol. 2023 Aug 17;13:1195814. doi: 10.3389/fonc.2023.1195814 (PMC10471184; doi:10.3389/fonc.2023.1195814)
Supplement: Supplementary file 1 [file Table_1.docx]

Supplementary Material

Constitutional mismatch repair deficiency syndrome with atypical features caused by a homozygous *MLH1* missense variant (c.1918C>A, p.(Pro640Thr)): a case report

Firas Akrout†*, Ahlem Achour†, Carli M. J. Tops, Richard Gallon, Rym Meddeb, Sameh Achoura, Mariem Ben Rekaya, Emna Hamdeni, Soumaya Rammeh, Ridha Chkili, Nada Mansouri, Neila Belguith, Ridha Mrad

^†^ These authors contributed equally to this work and share first authorship

*** Correspondence:** Firas Akrout: akrout.firas@gmail.com

# Supplementary Tables

Supplementary Table 1 Fragment length analysis of six short tandem repeat markers of microsatellite instability (MSI) in blood DNA samples of the father, mother, and patient, along with colorectal mucosa and tumor samples of the patient.

| Marker | Father blood DNA | Mother blood DNA | Proband blood DNA | Colorectal mucosa | Tumor colorectal tissue | Tumor cerebral tissue | Tumor parotid tissue | Tumor ampulla of Vater |
| --- | --- | --- | --- | --- | --- | --- | --- | --- |
| HSP110 | 167/167 | 167/167 | 167/167 | 164/167  (-3bp) | 164/167  (-3bp) | 162/167  (-4bp) | 164/167  (-3bp) | 164/167  (-3bp) |
| BAT26 | 181/181 | 181/181 | 181/181 | 179/180  (-2bp) | 179/180  (-2bp) | 180/181  (-1bp) | 177/180  (-4bp) | 178/179  (-3bp) |
| BAT25 | 152/153 | 152/152 | 152/152 | 150/152  (-2bp) | 150/152  (-2bp) | Not amplified | 148/152  (-4bp) | 147/152  (-5bp) |
| NER24 | 127/127 | 126/127 | 126/127 | Not amplified | Not amplified | 126/127 | 124/127  (-3bp) | 125/127  (-2bp) |
| NR21 | 112/112 | 110/111/112 | 110/112 | 108/110  (-2bp) | 108/110  (-2bp) | 110/112 | Not amplified | 109/111  (-1bp) |
| NR27 | 84/84 | 84/84 | 84/84 | 82/84  (-2bp) | 82/84  (-2bp) | 79/84  (-2bp) | 80/84  (-4bp) | 80/84  (-4bp) |
| MSI Score | 0 | 0 | 0 | 1.00 | 1.00 | 0.60 | 1.00 | 1.00 |
| MSI Status | MSS | MSS | MSS | MSI | MSI | MSI | MSI | MSI |

The observed fragment length for each MSI marker in each sample is shown. When calculating the base pair (bp) shift in marker fragment length (observed shifts are given in brackets), the fragment length in DNA from peripheral blood leukocytes (bDNA) was used as a reference. Markers were considered unstable if a shift in fragment length was observed compared to bDNA samples. An MSI score was calculated per sample, representing the proportion of markers that were unstable. An MSI score ≥0.3 classified a sample as having MSI, and an MSI score <0.3 classified a sample as microsatellite stable (MSS) . We found that the colorectal mucosa and all tumor tissues of the patient have MSI.
